# Towards circular phosphorus: The need of inter- and transdisciplinary research to close the broken cycle

**DOI:** 10.1007/s13280-021-01562-6

**Published:** 2021-05-19

**Authors:** Christian Stamm, Claudia R. Binder, Emmanuel Frossard, Philip M. Haygarth, Astrid Oberson, Alan E. Richardson, Christian Schaum, Oscar Schoumans, Kai M. Udert

**Affiliations:** 1 Eawag, Swiss Federal Institute of Aquatic Science and Technology, 8600 Dübendorf, Switzerland; 2 Laboratory on Human-Environment Relations in Urban Systems, EPFL ENAC IIE HERUS, 1015 Lausanne EPFL, Switzerland; 3 ETH Zurich, Research Station in Plant Sciences, Eschikon, 8315 Lindau, Switzerland; 4 Lancaster Environment Centre, Lancaster University, Lancaster, LA1 4YQ UK; 5 Group of Plant Nutrition, Research Station Eschikon, Institute of Agricultural Sciences, ETH Zurich, Eschikon 33, 8315 Lindau, Switzerland; 6 CSIRO, Agriculture & Food, PO Box 1700, Canberra, ACT 2601 Australia; 7 Chair of Sanitary Engineering and Waste Management, Bundeswehr University Munich, Werner-Heisenberg-Weg 39, 85577 Neubiberg, Germany; 8 Wageningen University & Research, Droevendaalsesteeg 3, 6708 PB Wageningen, The Netherlands; 9 Eawag, Process Engineering, 8600 Dübendorf, Switzerland

**Keywords:** Agriculture, Science-practice/policy interface, Wastewater

## Abstract

Phosphorus (P) is an essential element to all living beings but also a finite resource. P-related problems center around broken P cycles from local to global scales. This paper presents outcomes from the 9th International Phosphorus Workshop (IPW9) held 2019 on how to move towards a sustainable P management. It is based on two sequential discussion rounds with all participants. Important progress was reported regarding the awareness of P as finite mineable resource, technologies to recycle P, and legislation towards a circular P economy. Yet, critical deficits were identified such as how to handle legacy P, how climate change may affect ecosystem P cycling, or working business models to up-scale existing recycling models. Workshop participants argued for more transdisciplinary networks to narrow a perceived science-practice/policy gap. While this gap may be smaller in reality as illustrated with a Swiss example, we formulate recommendations how to bridge this gap more effectively.

**Supplementary Information:**

The online version contains supplementary material available at 10.1007/s13280-021-01562-6.

## Introduction

Phosphorus (P) is an essential element to all living beings. The increasing food demand for a growing human population, along with a globally greater share of animal products in human diets, will increase the demand on P fertilizers (Scholz et al. 2014; Elser and Haygarth 2021). They are produced from limited mineable rock phosphate deposits, which are located in few countries, resulting in a delicate geopolitical dependence of food production systems. At the same time, globally a large share of P contained in societal wastes such as urban wastewater is not reused for agricultural production. It accumulates in soils, is discharged to water bodies instead or stored in landfills (partially after incineration) (Schoumans et al. 2015; Powers et al. 2016).

This broken P cycle causes several problems (Elser and Bennett 2011). On the one hand, a valuable resource is wasted, on the other hand, some of the wasted P is lost from agriculture and from urban sources to water bodies. Untreated wastewater is a major P input into water bodies in many countries (Baum et al. 2013). Where a well-developed waste water treatment plants (WWTP) infrastructure exists, the P-load to standing waters is generally dominated by diffuse agricultural sources. However, discharge from WWTP may still significantly increase time-averaged P levels in streams causing ecological problems (Stamm et al. 2014).

In consequence, P has become a major factor in the global change exceeding planetary boundaries (Steffen et al. 2015) via eutrophication of aquatic systems and by threatening the biodiversity in terrestrial systems (Ceulemans et al. 2014). Climate change is expected to amplify P-related challenges. This is expected through increased losses from terrestrial systems due to more intensive rainfall events and flooding, or via increased P deficiency to crops related to adverse soil moisture conditions (Ockenden et al. 2016).

To address these issues and develop sustainable solutions, different scientific disciplines need to provide their specific understanding and insights. Communities of various research disciplines are indeed very active in this area. Accordingly, the research community in the field of agronomic and environmental aspects of P fertilization meets at different conference and workshops. One of them is the “International Phosphorus Workshop” (IPW) series that started in 1995 in Wexford (Ireland), discussing P losses from agricultural land to water (Tunney et al. 1997). The uniqueness of the International Phosphorus Workshops is the combination of research on terrestrial and aquatic ecosystems. The IPWs arose from the understanding that combating eutrophication of waters requires the proper understanding of biogeochemical, soil physical and hydrological mechanisms and processes underlying the terrestrial-aquatic P-continuum. The IPW series kept a strong focus on improving P use efficiency in agro-ecosystems. But it got more and more evident that minimizing agricultural P losses (Sharpley et al. 2015) cannot be achieved without tackling the problem of broken P cycles within the agricultural sector and between other sectors and agriculture (Leinweber et al. 2018). This insight developed from the fact that the simultaneous problems of P scarcity and excess are known for decades, many projects such as the European COST Action 869, SERA17 in the USA (http://sera17.org) and others have provided concrete measures for improvement (see, e.g., Schoumans (Ed.) et al. 2011) since many years, but the problems still remain. Obviously, substantial progress is still needed and key questions arise how to move forward.

The problem perception how to manage the P cycle from a local to a global level may differ substantially between stakeholders as will their preferred solutions. These characteristics are typical for wicked problems (Rittel and Webber 1973). One insight into handling them is the need to collaborate in research and practice across disciplines to consider different perspectives (Ulrich et al. 2013). Such collaboration may take different forms and we distinguish here disciplinary, interdisciplinary and transdisciplinary research. Interdisciplinarity is characterized by joint problem definitions across disciplines (Mobjörk 2010), transdisciplinary research describes a scientific co-production with practice.

To respond to this need and foster the inter- and transdisciplinary exchange, IPW9 (Zürich, 8.—12. July 2019), actively invited participants from natural, engineering and social sciences to discuss concepts, which drive research and use of P in our societies, to review the progress in knowledge and technology related to P management over the last years and to define questions for future research and action to solve P-related problems. The participating experts covered a broad range of scientific fields (forestry, agriculture, soil sciences, aquatic sciences, sanitation and waste management, political sciences, etc.).

To actively facilitate the interdisciplinary exchange among the participants two sequential workshops engaged them into a structured discussion to (i) identify important research questions from both a disciplinary and interdisciplinary perspective, and (ii) formulate ways and approaches to tackle these questions. This paper presents the main outcomes from these two workshops, consolidates and finally critically reviews them. It also compares the degree of interdisciplinarity and research orientation of the individual oral and poster presentations with those of the key questions resulting from these two workshops. This comparison provide insight into needs for fostering inter- and transdisciplinary exchange. Subsequently, the development of P recycling in Switzerland over the last decades is used as an example to illustrate how science and policy-making interacted in shaping practical solutions. Finally, the paper concludes with some recommendations to foster the transdisciplinary process and propositions for a future research agenda. They shall help improving P use efficiency and decreasing P losses from the local to global level.

## Approach

### Workshop organization

The IPW9 conference featured 187 individual oral and poster presentations (excluding key note lectures) and two sequential workshops organized on day 3 and 4 of the conference to jointly identify key questions for further research and promising research fields. About 170 out of the 220 IPW9 participants from 31 countries actively engaged themselves in these discussions.

The first workshop addressed the questions from a specific thematic perspective. To that end four topic groups were formed (Topic 1: P scarcity / optimizing P cycles, Topic 2: sourcing P fertilizers, Topic 3: efficient P use in agro- and forest ecosystems, Topic 4: environmental P problems). The participants joint the group of their research topic. Each group was led by a key moderator who was supported by 2–4 table hosts for guiding the discussions in split groups. For each topic, the same set of leading questions were addressed:

- Where did we make substantial progress over the last 5 to 10 years, where did we stall despite earlier expectations for progress?
- What are current and future key research questions in the field?
- What are the most promising methods or approaches for tackling these questions?

The key moderators and the organizing committee compiled the outputs of the workshop and derived key research questions for the subsequent interdisciplinary workshop (see Results section). This second workshop aimed at an interdisciplinary and transdisciplinary review of these key questions. Accordingly, participants were assigned randomly to four groups. Participants identified synergies and linkages between the thematic research questions, pointed out gaps and provided new ideas based on the inter- and transdisciplinary view. The transdisciplinary character was not very strong though given the limited participation from outside academia.

The outcome of the second workshop was again compiled and synthesized by the key moderators and the organizing committee such that the main outcome could be presented and discussed in a plenary session on the final day of the conference. For this article, the author team consisting of the workshop moderators and organizing committee has scrutinized the workshop outcome and the discussion points in the plenary.

#### Characterizing the IPW9 key questions and abstracts of the individual presentations

To compare the content of the individual oral and poster presentations by the participants during IPW9 with the key questions jointly developed during the two workshops, the abstracts of the individual presentations and these key questions were characterized (see SI for an annotated abstract) regarding (i) the degree of inter- and transdisciplinarity and (ii) the gradient between basic research focusing on process elucidation and implementation of full-scale solutions (called Research orientation) (see Fig. 2). To that end, the gradients along these two dimensions were operationalized by number of disciplines involved, the spatial and temporal scales of investigations and the knowledge type(s) (i.e., system, target and transformation knowledge; (Proclim/CASS 1997) (see below).

The degree of interdisciplinarity was assessed considering the number of disciplines (agronomy, political sciences, engineering, etc.), number of environmental compartments (soil, water, waste, etc.), number of research domains (natural, social or engineering sciences, humanities), and the number of socio-economic sectors (e.g., agriculture, forestry, environment, see SI Table S1). Some of these factors revealed correlations (number of disciplines and sectors), but including all of them added descriptive differentiation.

For each of these factors, an attribute list (Table S1, S2) was compiled to cover the contents of the submitted abstracts and the key questions. These factors were used to define the degree of interdisciplinarity (*D*_disc_) as follows:

1 $$D_{{{\text{disc}}}} = N_{{{\text{compartments}}}} + N_{{{\text{disciplines}}}} + N_{{{\text{domains}}}} + N_{{{\text{sectors}}}}$$

where *N*_*i*_ represents the number of compartments, disciplines, domains, and sectors per abstract or key question, respectively.

The research orientation was assessed with the three factors spatial dimension, temporal dimension and knowledge type. For each of these factors, the attributes were rated the higher the large, longer and more inclusive they are (weights $v_{{{\text{space}}}} ,v_{{{\text{temp}}}} ,v_{{{\text{type}}}}$). A study at the continental scale for example got a higher value than one at the plot scale for the spatial dimension (see Table S2). Finally, for each abstract or key question the maximum values along the three dimensions (*D*_orient_) were summed up:

2 $$D_{{{\text{orient}}}} = \max \left( {v_{{{\text{space}}}} } \right) + \max \left( {v_{{{\text{temp}}}} } \right) + \max \left( {v_{{{\text{type}}}} } \right)$$

The entire procedure is illustrated with an annotated abstract in the SI (Table S3).

## Results and discussion

### Output from the workshops

In the following, we summarize the most important topics discussed during the workshops. The contents reflects the contributions by the participants as perceived and summarized by the key moderators of the respective workshops.

#### Topic 1: Phosphorus scarcity/Optimizing Phosphorus cycles

The worldwide food production highly depends on the input of P as one of the macronutrients for plant growth and as a feed additive for livestock production. However, the worldwide phosphate rock reserves of good quality and which can currently profitable mined, are limited and estimated at 69 000 Tg P while the world mining production in 2019 was 240 Tg P (USGS, 2020). Furthermore, about 70% of the reserves are located in Western Sahara and Morocco. Europe has no significant P reserves and highly depends on P import. During the discussion it became clear that over the last two decades the awareness of Europe’s dependency on P import has significantly increased, and became a main topic in Europe’s Circular Economy strategy (EC 2015). Furthermore, there is agreement that the 5R stewardship (Withers et al. 2015) is of high importance as basic principle: Re-align P inputs, Reduce P losses, Recycle P in bioresources, Recover P in wastes, and Redefine P in food systems. However, some additional notes were given. First, the focus should not be solely on P but on C, N and P in an integral way. Also N is highly linked to organic sources and furthermore, organic matter is needed to maintain a good soil structure and soil fertility/quality status. Secondly it was mentioned that the European agricultural production system became highly specialized, and the interlinkage between crop and animal / meat production disappeared. This results in imbalances in terms of P surpluses and deficiencies, not only at regional scale but also between nations. This caused severe environmental pollution in areas with high P surpluses, since nutrient application rates with manure were often much higher than the nutrient requirements of the crops. Finally, it was mentioned that to date there is no European strategy or legislation for P in analogy to nitrogen (EEC 1991). The general conclusion was that there is a “system-based approach” needed to optimize P cycling. However, no real framework was defined due to different ideas about how to (a) include different scales (field – farm – region – national – EU regions), (b) define acceptable P rates (agronomical requirements versus environmental goals) and (c) include the role of P accumulation. Only few European countries have implemented P legislation from an environmental point of view prescribing for example application standards for different soil P status (Amery and Schoumans 2014). It would be worthwhile to develop such a real “system based P approach” as standardized principle for the use of P within agriculture systems. A broad implementation will give an important incentive to reuse and valorize available P sources in a more efficient way and give a better closure of the P cycle.

#### Topic 2: Sourcing Phosphorus fertilizers

The discussions on the subject of "Sourcing P fertilizer" initially covered the substantial progress in the recent years. In municipal wastewater treatment, the "technology readiness level" of many P recovery technologies has increased significantly, so that a large-scale technical realization should soon be possible (Schaum 2018). This is mainly due to a perceived need to close the P cycle and to slow down the rapid depletion of mineral phosphorus resources. However, for wastewater engineers in particular, preventing eutrophication by high P discharges into receiving waters remains the main target for P removal. The discussions on possible P scarcity (Scholz and Wellmer 2013) strengthened the cooperation between administration, agriculture and wastewater management, which is important to develop optimal strategies for closing the P cycle. This also made it possible to open up new waste streams for P recovery, e.g. meat-and-bone meal or excess manure that cannot be recycled directly as organic fertilizer. These factors have led to new regulations aimed at implementing P recovery, for example in Switzerland and Germany.

Even if developments in the field of P recovery have made great progress, a decisive step towards large-scale implementation is now required. The integration of P recovery in a holistic approach for P management was identified as a key challenge. During the last years, a high technology readiness levels has been achieved. Now, it is necessary to develop cost/business models, which target the needs of the end user—the fertilizer manufacturers and farmers—of the recovered P. Product quality and the associated marketing play an important role. Besides the P availability, impurities and their possible negative effect on soil and crops determine the product quality. The extent of P recovery and the quality of the products could be increased to further develop and optimize the recovery from alternative sources such as urine and feces collected at their sources.

While developing optimal technologies and approaches for P recovery, the workshop participants also expressed the need to widen and strengthen the global trans- and interdisciplinary network. One important goal is the development of appropriate technical standards and regulations. One step in this direction is the recently developed private standard on recycling products from dry toilets (DIN 2020). Last but not least, P recycling must be seen in a larger context, including the sustainable use of all resources involved in agriculture and waste management. Future changes due to climate change should be considered but also the challenges of soil depletion and eutrophication. The further development of LCA-type analyses and decision-making tools can make an important contribution to a holistic approach.

#### Topic 3: Efficient Phosphorus use in agroecosystems

In intensive agricultural systems, including those with a high density of animals, over-application of fertilizer and excessive return of P to soil through manures remains a significant issue. High inputs of P to meet demand for productive crop yields has resulted in a widespread accumulation of P in many soils. Better utilization of legacy P and avoidance of continued accumulation of P is a key target for enhanced efficiency and improvement of the environment. Elsewhere, and especially in developing countries, the need for improved P efficiency is driven by a need for P inputs to overcome P deficiency in soil and its limited availability to crops. This need is further exacerbated by a run-down of endogenous soil P reserves and often by a limited access to reliable and effective sources of P fertilizer.

Considerable global research effort has been undertaken to address the efficiency of P use in agricultural systems. While our understanding of soil–plant and environmental interactions of P has greatly improved, the outputs of this research has had varying success in delivering widespread benefit. As recognized by this thematic workshop, demonstrable gains have been made in some areas, which include; (i) improved crop management and agronomic practice, with more targeted application of P fertilizers to meet plant needs; (ii) new fertilizer technologies, including the development of alternative products, formulations and controlled release products, together with greater considerations of 4R stewardship (i.e., right product at the right rate, right time with right placement (Johnston and Bruulsema 2014)); (iii) wider recognition and application of biologically based approaches to P mobilization, including inoculants, mycorrhizas, companion/cover crops and inter-cropping and (iv) more P efficient plants (Richardson et al. 2011). Across various plant species, germplasm with differential capacity for P-acquisition have been identified, as have plants with altered P-utilization efficiency. Nonetheless, there are only limited examples where ‘new’ germplasm has been practically deployed, with exception perhaps of cultivars of common bean and maize for use on low-P soils (Lynch 2019). Likewise, genetic modification and use of genetic markers for ‘P-efficiency’ traits, have not translated to practical use in agricultural systems.

The thematic workshop identified and prioritized a number of key opportunities and challenges: (1). Further need to expand and promote grower and industry awareness with respect to ‘Good Agricultural Practice (GAP)’ for P efficiency, with greater understanding of production targets, system requirements and environmental flows. This includes the provision of more reliable information and demonstrable and quantifiable benefits of ‘new’ technologies and ‘alternative’ practices. (2) More widespread application and capture of ‘P value’ from waste streams. This includes practical assessment of industry-wide opportunities along with realistic understanding of benefits of P recycling as viable fertilizer replacements and/or supplement options from a range of perspectives (agronomic, economic, environmental and societal). (3) Greater consideration and knowledge of the longer-term contribution of P as a ‘systems component’ and its integration with other biogeochemical cycles (e.g., C and N) and climate-environment based models across farms, landscapes and global systems.

#### Topic 4: Environmental Phosphorus impacts

The discussions were framed around the effects of P on undermining water quality. Therefore it was fitting that IPW9 took place in Zurich, the birthplace of Richard Vollenweider, the ecologist who was eminent in elucidating the role of P as an environmental pollutant (Vollenweider 1968). Even 50 years after this seminal work it was the overall position of IPW9 that P continues to be a significant threat to global water quality. Within this context, the contemporary research priority questions were: (1) What will happen to the P cycle and subsequent environmental problems with climate change? (2) How can we achieve an appropriate systems perspective to understand the environmental problems?, and; (3) How do we integrate socio-economic perspectives into understanding and mitigating the environmental problems?

The recognition that climate change is bringing about a shift in the behavior of P transfer from land to water, was the emerging priority research-need. Climate change will have all sorts of, as yet, unknown effects on the soil-catchment-water biogeochemical systems that will have consequences on the mobility of P through the environment (Forber et al. 2018). For example, there are unknowns about the changes in soil temperature and wetting and drying effects relevant for mobilizing P from soil (Blackwell et al. 2009, 2010). Also changes in rainfall patterns will have important consequences (Ockenden et al. 2016). Modeling predicts for ca. 30% increases in P transfers from land to water later this century, causing environmental problems (Ockenden et al. 2017). There are similar concerns and uncertainties to be evaluated in respect to behavior of P in water bodies (Robertson et al. 2016; Yankova et al. 2017). Moreover, there is a widening appreciation of the potential longer-term effects of P on environmental problems at the ocean margin and, potentially, even longer-term and widespread ocean anoxia (Watson et al. 2017).

The second priority question was how we can achieve an appropriate systems perspective to understand environmental P problems. Critically, the link between source and impact via the P transfer continuum (Haygarth et al. 2005) may span thousands of kilometers. The workshop also acknowledged issues around the delays in the response of environmental system, sometimes called ‘legacy’ P concept (Jarvie et al. 2013; Sharpley et al. 2013; Haygarth et al. 2014). It is this ‘disconnection’ in time and space that contributes to our challenges in understanding of the problem (Powers et al. 2016), and thus the need for an appropriate systems perspective to understand the spatio-temporal complexities linking environmental P problems.

The third and final priority question was how do we integrate a socio-economic perspective into understanding and mitigating the environmental problems? The discussion identified the need for a stronger P ‘narrative’ that reaches out to stakeholders including the public, authorities and industry. The need to move towards a circular economy was a particular priority area, which it was argued, would help reduce P problems. One example from Ireland (Macintosh et al. 2019) is an exemplar of working towards a circular economy that has successfully seeded and built the Irish Nutrient Sustainability Platform https://nutrientsustainability.ie/#.

#### Condensed outcome and critical review

**Fig. 1:**
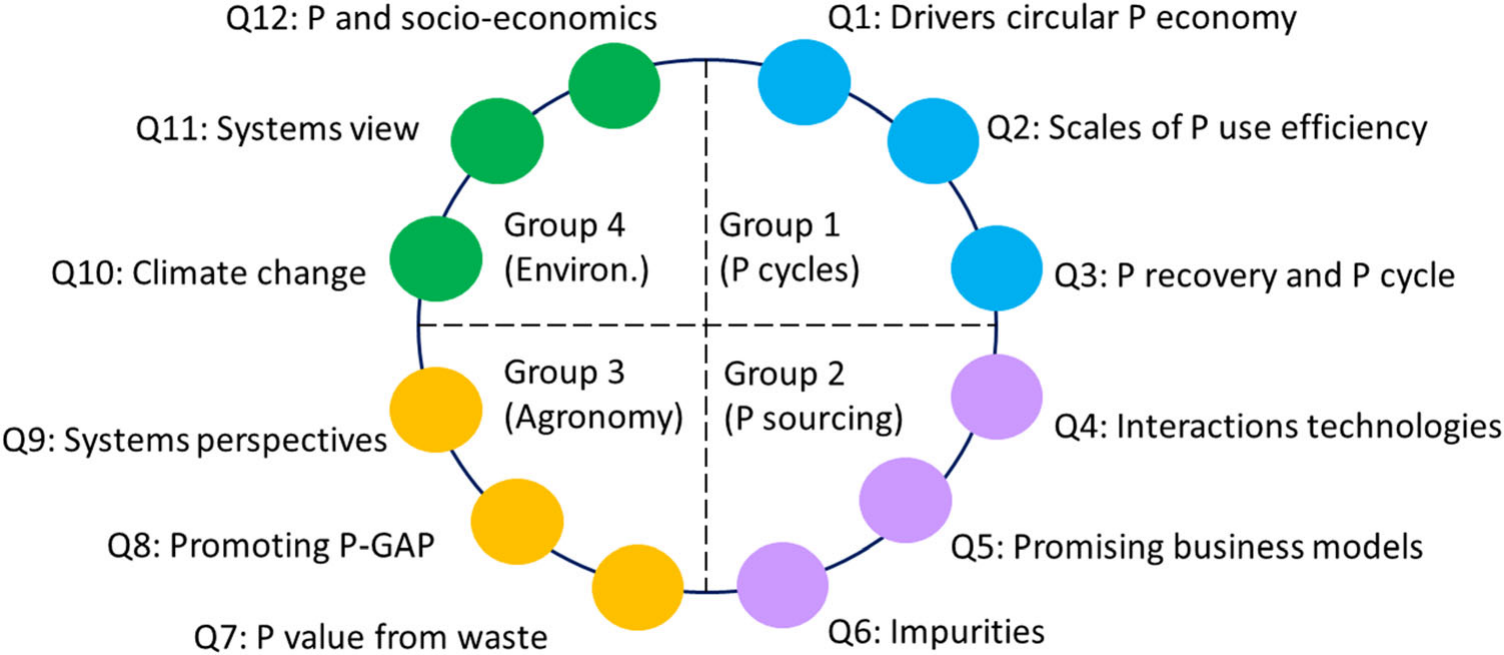
Key questions/topics resulting from the four thematic workshops. GAP: Good Agricultural Practice

Overall, the outcome of the thematic workshops was consistent in that they shared a number of general themes (system view, circular P economy, Fig. 1). These commonalities were complemented by specific aspects that were mentioned only by one group. During the following interdisciplinary workshop, the discussion was refined and links between the thematic perspectives were clarified. In the following, we highlight some of the aspects that emerged from the second round of workshops.

From an agronomic point of view, concepts of P use efficiency were thus far mostly limited to fertilizer P. However, it was noted that best management practices should consider the environmental, agronomic and socio-economic context of a region. They should also include recycling of P from waste streams such as residues from food production, excess manure or municipal wastewater, and consider the legacy P in soils and sediments. Several groups proposed integrated catchment scale studies that look at all possible P sources, including urban waste, the fate and transport of P within an agroecosystem, and the socio-economic factors influencing P recycling. The expectation was expressed that results of such integrated studies would allow elaborating more balanced best management practices. These practices must also consider linkages to cycles of other elements, especially C, N or trace elements.

In all workshop groups, climate change was perceived as being an underrated factor related to P problems and the proposed solutions. While it is certainly true that consequences of climate change on the P cycle must be considered, other global environmental issues, such as eutrophication and soil P-depletion are more strongly related to the anthropogenic P use (see, e.g. Steffen et al. 2015). For all these issues, workshop participants emphasized the need for a global perspective to address them. Most workshop participants came from regions of the world where excess determine the scientific perception of P. However, in many regions of the world, sufficient P supply is the preeminent problem. Actually, truly global P networks are missing, which would help to gain a balanced view of P issues in different regions of the world.

Frequently, participants emphasized the importance of considering socio-economic factors for sustainable P recycling from waste to soil and to get the private sector involved. In this context, the (perceived) science—practice/policy gap was a recurring theme. Two major deficits were mentioned: first, missing reach out of the scientific community to the broader public, and second, lack of awareness and deeper understanding of P problems to gain higher acceptance for drastic measures to address the issues.

Overall, during both rounds of workshops, many participants took a broad view on the P issues and proposed to follow a wide systems perspective for analyzing the situations and circular P economy was a frequently concept put forward as a solution. Interactions with stakeholders across sectors was considered essential for moving forward.

**Fig. 2:**
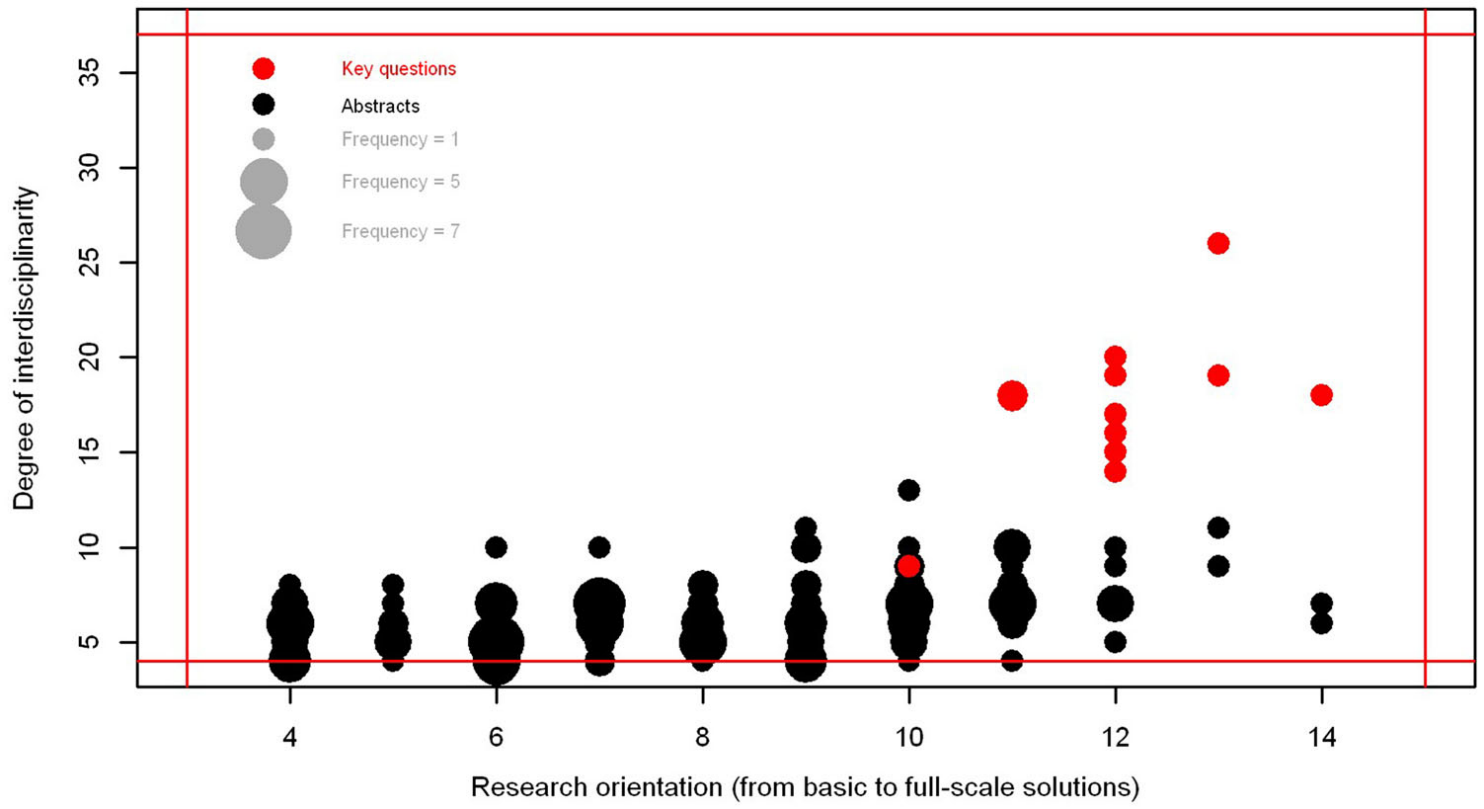
Mismatch between individual research niches as expressed in the submitted abstracts (black circles) and overarching objectives formulated in the 12 key questions (Fig. 1; red circles) evaluated along the two dimensions *Research orientation* (*x*-axis) and *Inter- /Transdisciplinarity* (*y*-axis) according to Eqs. 1, 2. The circle size is proportional to the frequency of items (abstracts, key questions) with the respective values. The red lines indicate the minimum and maximum values possible along each dimension

This broad view and the key questions identified during the thematic workshops contrast with the predominantly disciplinary research presented by the participants during the conference (Fig. 2). While the 12 key questions are all applied and are highly interdisciplinary, many presentations were rather on the side of basic research in the respective fields. There was a considerable number of oral and poster presentations that were practice-oriented but they generally fall short with regard to interdisciplinarity as compared to the key questions. This was mainly due to larger numbers of compartments, disciplines, and sectors considered by the key questions (see Fig. S2). This comparison suggests that the participants see the need for a more inter- and transdisciplinary research agenda that includes several economic sectors. The actual projects on which they report, however, seems not to provide sufficient opportunities to realize the desired holistic approaches.

### Moving towards change: examples of obstacles to overcome

There was a broad agreement among participants that closing the P cycle across sectors and spatial scales has to be emphasized more. However, many questions remained open as to how close this cycles. Repeatedly, it was mentioned that one has to better bridge the science-practice/policy gap to convince stakeholders to do “the right thing”. Because this aspect was considered so important, we use here the history of P use and recycling from the urban waste stream into agriculture in Switzerland during the last 40 years to illustrate some features of the science-practice/policy interface. It demonstrates the intricate and complex interactions of many societal processes and the time horizons typical for such socio-political and scientific processes. Finally, it also illustrates the role of scientific results in shaping the process and the implementation of the solution.

#### Phosphorus recycling and reuse from the Swiss wastewater stream

This section shows (i) how fundamental changes in P recycling from wastewater streams in Switzerland in the past were triggered by crises that were not directly related to P and the related interactions between practice, policy and science, and (ii) that in the (Swiss) policy discussion it is not possible to isolate the P issue from other topics. The historic development can be described as a sequence of marked paradigm shifts triggered by events not necessarily related to P recycling.

*The no-recycling paradigm*. Previous to the build-up of the WWTP infrastructure in the 1970s to1980s, there was no recycling of urban P losses in sewage to agriculture during the twentieth century. The WWTPs were constructed to clean water bodies, not to bring P back into the farming system.

**Fig. 3:**
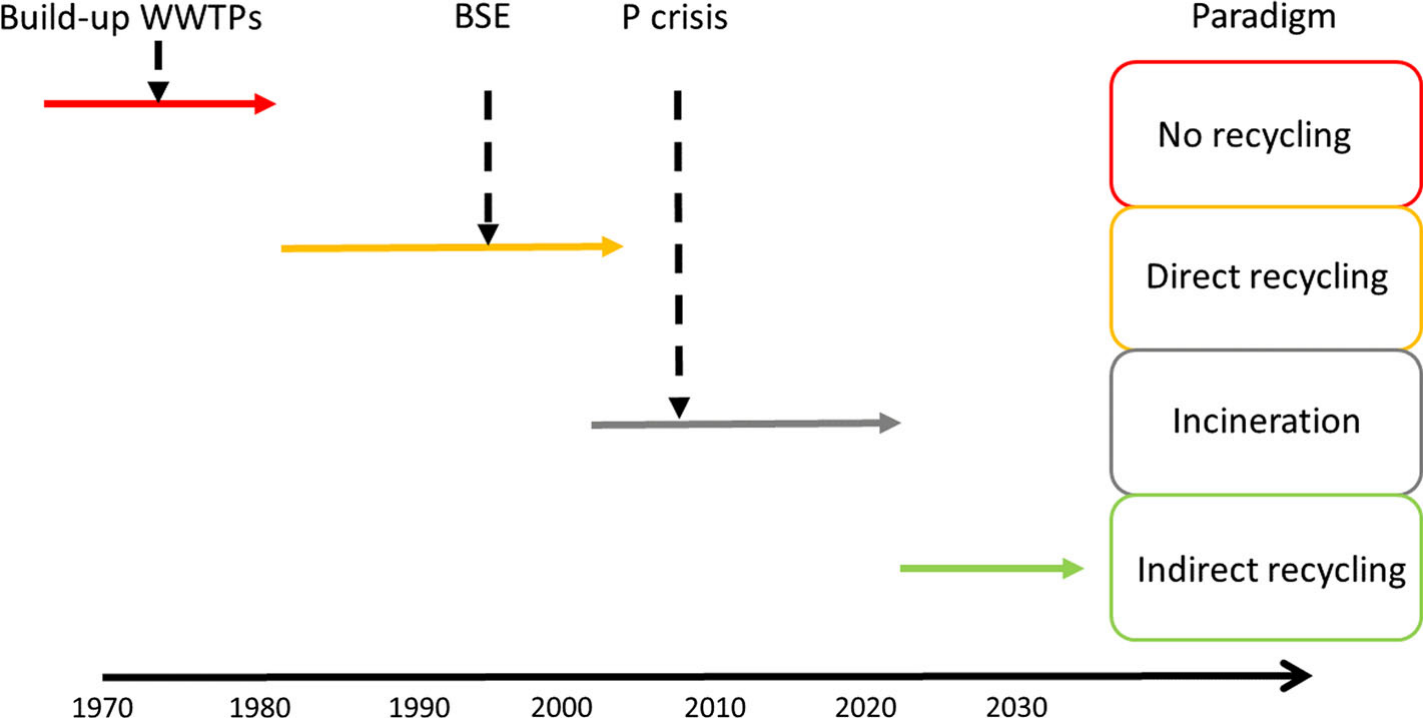
Time line of the paradigm shifts in P recycling in Switzerland (see also Mehr et al. (2018)). BSE: Bovine Spongiform Encephalopathy, WWTP: Wastewater Treatment Plant

*The direct recycling paradigm.* Phosphorus fluxes in waste waters is the most important P flux in the Swiss society outside of agriculture (Binder et al. 2009). Once the WWTP infrastructure existed, policies and practices had been developed and implemented to recycle sewage sludge in agriculture. In 1999, 55% of the sludge produced in Switzerland were recycled in this manner (Candinas et al. 1999). To organize this recycling procedure, cantons implemented services to optimize the contact between sludge producers and farmers, and to organize sludge analyses as well as soil analyses so that sewage sludge could be added without triggering excessive nutrient inputs and without violating the law on toxic substances in the environment (see, e.g. Bolliger (2007)). The practice of sludge recycling seemed therefore well established and sound (Fig. 3).

*The incineration paradigm.* The outbreak of bovine spongiform encephalopathy (BSE) in Switzerland in the 1990s, however, led to the end of the established system of sewage sludge recycling.

The possible link of BSE in cows and the BSE variation of the Creutzfeldt-Jakob disease in humans (Heim and Kihm 1999) had a large echo in the Swiss media. This triggered a distrust of consumers in agricultural products, and led farmers’ associations and food retailers to request a ban on sewage sludge recycling in agriculture (DETEC 2002). The Swiss government banned the use of sewage sludge in agriculture, leaving a transition period until 2005 (DETEC 2002). Since then all sewage sludge has been incinerated and the ashes have been deposited in special landfills because of the high heavy metal contents of sludge.

*The indirect recycling paradigm.* Only few years later, the topic of P recycling received a renewed interest from policy-makers, waste managers and scientists. This interest was related to the realization that the amount of “easily” mineable phosphate rock was finite, and that most of the known reserves were located in limited number of countries (Cordell et al. 2009; Van Kauwenbergh et al. 2013). The sudden and strong increase in the prices of P fertilizers in 2008 worldwide aggravated the perception that P fertilizers would become soon rare. Although discussions on scarcity of P reserves have been going on since the nineteenth century (Ulrich and Frossard 2014), and that the increase in the prices of P fertilizers of 2008 was not linked to phosphate rock scarcity (Scholz et al. 2014, p. 34), the fear that mineral P fertilizers would become less accessible was heard by policy-makers and led them think more about P recycling. This approach was embedded in a more global approach on circular economy both in Switzerland (DETEC 2015) and in Europe (Rizos et al. 2017). This led to a complete revision of the Swiss ordinance on waste (OTD 2015) declaring that P must be recovered from waste streams and then recycled.

This new paradigm change boosted research to recover P from wastes by Swiss institutions. Sewage ashes have attracted most attention representing the large majority of the P from the wastewater stream (Binder et al. 2009). A major obstacle was to find an appropriate market for these new P fertilizers. Finally, a process (the Phos4life process) was developed to produce pure H_3_PO_4_ instead of a fertilizer (Schlumberger 2019). This is acceptable both from a technical and a financial point of view. By producing H_3_PO_4_, the product can be used not only for agronomic purposes but also as food additive or for other industrial processes (Younes et al. 2019). Accordingly, the process will be implemented in a plant, which will be operational in 2025 and which will treat 30 000 t ashes/year (Schlumberger 2019).

In summary, the sequence of paradigm shifts illustrates that science and research played an important role in identifying problems, their causes and in developing new solutions to societal needs. But the scientific impact on actual political and societal decisions were strongly molded by public perception of risks and opportunities, which varied over time as did the scientific state of the art (Mehr et al. 2018). The example also illustrates that it is not possible to consider P in isolation. P recycling is more a consequence of other large debates like food safety or circular economy. It is the role of the P community to bring P issues onto the table in these larger debates.

### Closing the science – to – practice/policy gap for solving Phosphorus issues

The example described above illustrates that scientific findings did influence the political decision-making in the past. This seems to contradict the gap between science, policy-making and practice mentioned by many participants during the workshop. This apparent contradiction may be partially explained by unrealistic expectations on the side of researchers how scientific results find their way into the political decision-making process and the expectations that this process shall always lead to the outcomes preferred by scientists.

Another part of the explanation may be the observation that there is not a gap per se but that the interface between science and practice and science to policy is not optimally shaped. Actually, the limited participation from outside academia at IPW9—as mentioned above—illustrated this deficiency directly. This is unfortunate since Binder et al. (Binder et al. 2020) found that the benefit of scientific results was perceived to be higher the more interactions took place between practice and science. Higher interaction lead to higher trust and the feeling that one can support the results and that their applicability is higher (Binder et al. 2020). Therefore, we summarize a number of recommendations brought forward during the workshop to improve on the science—practice/policy interactions.

*Individual researchers* have a direct influence contributing to solve P problems through their own research and education activities. They can actively look for research calls where they can contribute with their disciplinary expertise to find answers for overarching topics (Fig. 1). Furthermore, they could involve stakeholders in (i) jointly defining the research question (Norström et al. 2020) (ii) providing data and feedback during the research process, and (iii) discuss the relevance and implication of the results (Binder et al. 2020). By including such inter-and transdisciplinary aspects into education of students, researchers may exert long-lasting effects by providing not only adequate factual knowledge but also by enhancing the skills of the future generation to deal with problems at the science-practice interface.

Additionally, scientists play an eminent role in organizing scientific exchange through conferences and workshops such as the IPW series. In this role, scientists may think beyond the classical formats and include new forms such that (part of a conference) aims at a targeted exchange with stakeholders. This requires appropriate formats regarding timeframe and contents to meet stakeholder needs and interests.

Individual researchers are strongly influenced in their activities by the financial means available. Accordingly, *research institutions* and *funding agencies* play an essential role in providing opportunities to engage in such projects. On the one hand, money should be allocated for practice partners to contribute to the shaping of the research question and for workshops to give feedback (Binder et al. 2020). They can also support networking among researchers and practitioners to provide research partners and facilitate the implementation of the results by including the whole value chain (Fritz et al. 2019). On the other hand, the engagement of scientists in transdisciplinary collaborations has to be reflected in the way researchers are evaluated during their scientific career (Klein 2008; Dilling and Lemos 2011).

It is evident though that steps undertaken by the research communities and institutions to close the science-practice/policy gap are simultaneously accompanied by the openness by other stakeholders. A*uthorities, industry or NGOs* need to engage in joint activities and to also invest the necessary resources such as time to bring such collaboration to life. Joint projects may be very effective in bridging the gap, especially when they center around a problem of shared interest. However, finding the relevant partners at the interface between science, practice and policy may be difficult without previous networks. *Intermediary organizations* may provide platforms where different stakeholders meet and exchange because their activity address the different wishes and needs (e.g., networking among business partners, knowledge exchange, etc.) of stakeholders at the same time.

## Supplementary Information

Below is the link to the electronic supplementary material.

Supplementary file 1 (PDF 199 kb)
