# Supplementary material for: Towards circular phosphorus: The need of inter- and transdisciplinary research to close the broken cycle
Source: Ambio. 2021 May 19;51(3):611–22. doi: 10.1007/s13280-021-01562-6 (PMC8800955; doi:10.1007/s13280-021-01562-6)
Supplement: Supplementary file 1 — (PDF 199 kb) [file 13280_2021_1562_MOESM1_ESM.pdf]

Ambio

Electronic Supplementary Material

This supplementary material has not been peer reviewed.

Title:

Towards circular phosphorus: the need of inter and trans-disciplinary research to close the broken cycle

Factors and attributes for evaluating the abstracts and key questions along the two dimensions “Interdisciplinarity” and “Research orientation”.

Tab. S1: Factors and attributes for the dimension “Inter-/Transdisciplinarity”.

**Factor**	**Attributes**
Number of compartments (comp)	Air, biota (incl. crops), feed, fertilizer, food, sediment, soil, technical infrastructure, waste/manure, water
Number of disciplines (disc)	Agronomy (incl. aquaculture), atmospheric sciences, behavioral sciences, (aquatic, soil) biology, (aquatic, soil) chemistry, economics, food sciences, forestry, (physical) geography, hydrology, (aquatic, soil) physics, political sciences, process engineering, soil science (general)
Number of domains (domain)	Engineering (incl. agronomy), humanities, natural sciences, social sciences (incl. economics)
Number of sectors (sect)	Agriculture/aquaculture, environment, fertiliser industry, food system, forestry, institutional sector, waste processing

Tab. S2: Factors and attributes for the dimension "Research orientation".

**Factor**	**Attributes**	**Attribute weights (for the sequence of respective attributes**
Spatial scale (space)	Lab/pot scale, plot, field, catchment, region, national, continental, global	v_{space}: 1, 2, 3, 4, 5, 6, 7, 8
Temporal scales (temp)	Short-term (weeks/days), medium term (months - 1 yr), long-term (> 1 yr)	v_{temp}: 1, 2, 3
Knowledge type	Systems knowledge, Target knowledge, Transformation knowledge	v_{type}: 1, 2, 3

Illustration of an analysed abstract

The abstract below was selected such as to provide an instructive example of the procedure.

In the text, key words pointing to the elements of the dimensions Inter-/Transdisciplinarity (compartments, disciplines, domains, sectors) and Research orientation (spatial scale, temporal scale, knowledge type) are highlighted with according to the colour code given in this description. However, we also considered implicit information, on the domains for example (here: natural and engineering sciences).

The quantitative evaluation for this abstract is illustrated in Tab. S3 below.

Abstract text:

The Flemish Land Agency (VLM) initiated a research project (2015-2018) in order to select the best soil P test method, to set the target soil P availability for both agriculture and environment,
and to formulate fertiliser and management recommendations for sustainable P use.

Six soil P tests were evaluated: (1) extraction with ammonium lactate and acetate at pH 3.75 (P-AL, Egnér *et al.*, 1960); (2) extraction with 0.5 M $NaHCO_3$ (P-Olsen); (3) extraction with 0.01 M $CaCl_2$ (P-$CaCl_2$); (4) extraction with ammonium oxalate (P-Ox), with possibility to calculate the phosphate saturation degree (PSD, 5) and (6) diffusive gradient in thin film technique (DGT). In general, no large differences in soil P test performance for explaining yield (losses) were observed in 11 long term field studies across Europe (Nawara et al., 2017) and in a greenhouse P depletion trial (Nawara et al., 2018).
The capacity of the soil P tests in estimating soil P leaching losses were evaluated by a soil column leaching experiment, in which the PSD slightly outperformed P-AL and P-$CaCl_2$. P-Ox performed worst for both yield and P leaching estimations. Taking the method performance and the familiarity into account, it is reasonable to keep using the P-AL method in Flanders.

It was investigated which P-AL values are acceptable for both agriculture and environment. First of all, field trials were studied to look for the minimum P-AL value necessary for acceptable crop yields. A Mitscherlich fit revealed that for all crops together and at optimal soil pH, a P-AL value of 110 mg P/kg is necessary to reach 95% relative yield (yield compared to the maximal yield in that field). Secondly, it was investigated which P-AL in the topsoil is related to 25% PSD in the soil profile, limiting the P concentration leaching to the ground water to 0,1 mg ortho-P/L (P standard for surface waters in Flanders). This maximum P-AL acceptable for the environment is

160 mg P/kg. Consequently, the agro-environmental target zone for Flanders is between 110 and 160 mg P/kg measured in the topsoil.

On soils with P-AL values lower or higher than this target zone respectively positive or negative soil P balances (= inputs by P application minus outputs by crop removal) should be applied. A multiple linear regression with data from
33 long term field trials revealed that the higher the initial P-AL value, the more P fixation in time and the larger the net P-AL change per kg P added or removed from the soil.
To stay in the target zone, a small positive soil P balance is necessary. Interestingly on average only 27% of the net amount of P added or removed is measured as change in the P-AL fraction of the topsoil.

The results of the project will nourish the Flemish legislation and recommendations regarding P fertiliser strategies (quantities and management) are issued to the farmers.

Tab. S3: Summary of the text analysis of the example abstract

**Factors / Dimensions**	**Value**	**Identified elements**
Compartments	3	Biota, soil, water
Disciplines	3	Agronomy, soil chemistry, soil physics
Domains	2	Engineering sciences, natural sciences
Sectors	2	Agriculture, environment
**Interdisciplinarity**	**10**	
Knowledge types	3	Target knowledge, Transformation knowledge
Spatial scales	3	Field scale
Temporal scales	3	Long-term
**Research orientation**	**9**	

Comparison of the research orientation between key questions and abstracts

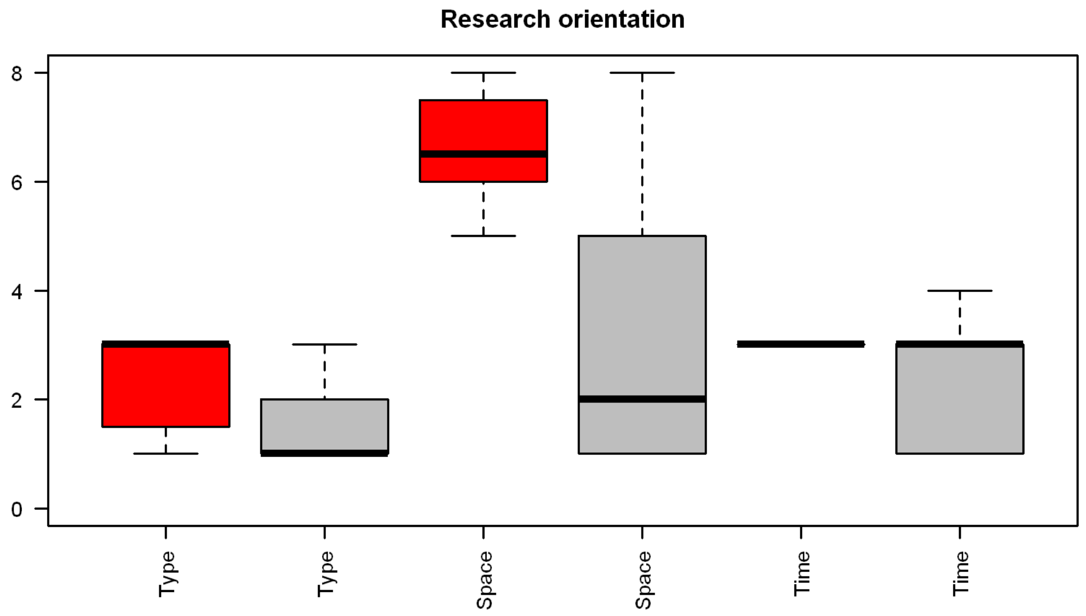

created with Rscript_IPW9_Anal_Abstracts_v20200409.R Thu Dec 10 10:42:17 2020

Fig. S1: Comparison of the values of the key questions (red) and the abstracts (grey) for the three factors (Knowledge type (Type), special dimension (Space), and temporal dimension (Time)) composing the degree of research orientation.

Comparison of interdisciplinarity between key questions and abstracts

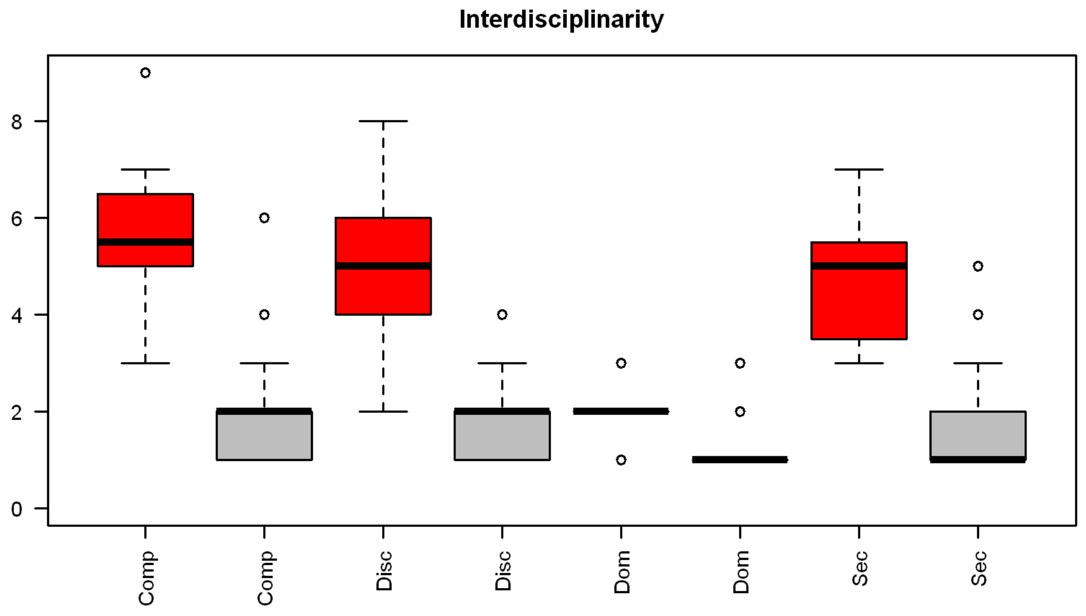

created with Rscript_IPW9_Anal_Abstracts_v20200409.R Thu Dec 10 10:32:19 2020

Fig. S2: Comparison of the values of the key questions (red) and the abstracts (grey) for the four factors (number of compartments (comp), number of disciplines (disc), number of domains (dom), and number of sectors (sec)) composing the degree of interdisciplinarity.
